# Adenoid Cystic Carcinoma of the Breast: Clinical and Radiological Findings

**DOI:** 10.3390/diagnostics16121869

**Published:** 2026-06-16

**Authors:** Jungmin Hwang, Boo-Kyung Han, Eun-Sook Ko, Ji Soo Choi, Jeongmin Lee, Haejung Kim, Myoung Kyoung Kim, Hyunwoo Lee, Eun Young Ko

**Affiliations:** 1 Department of Biological Sciences, University of Illinois at Chicago, 1200 W Harrison St, Chicago, IL 60607, USA; 2 Department of Radiology, Samsung Medical Center, Sungkyunkwan University School of Medicine, 81 Irwon-ro, Gangnam-gu, Seoul 06351, Republic of Korea; bkhan@skku.edu (B.-K.H.); es.ko@samsung.com (E.-S.K.); jisoo.choi@samsung.com (J.S.C.); jmlee328@gmail.com (J.L.); hjkim220@naver.com (H.K.); myoungkk@gmail.com (M.K.K.); 3 Department of Pathology and Translational Genomics, Samsung Medical Center, Sungkyunkwan University School of Medicine, 81 Irwon-ro, Gangnam-gu, Seoul 06351, Republic of Korea; hwpatho.lee@samsung.com

**Keywords:** adenoid cystic carcinoma, breast cancer, triple-negative breast neoplasms, neoadjuvant chemotherapy, Ki-67

## Abstract

**Background/Objectives**: Breast adenoid cystic carcinoma (ACC) is a rare tumor with limited data on imaging features and treatment response. This study investigated the clinical and radiological characteristics of ACC of the breast. **Methods**: Patients with ACC who underwent surgery at our institution between February 2010 and December 2023 were included. Clinical characteristics, biopsy and surgical pathology findings, and follow-up outcomes were reviewed. Preoperative mammography, ultrasound (US), and MRI findings were analyzed. **Results**: Twenty-eight women (mean age, 57 ± 8 years) were identified. Half presented with palpable masses, and the remainder were detected on screening. Percutaneous biopsy was performed in 27 patients, correctly diagnosing ACC in 18 (66.7%), whereas 9 (33.3%) were misdiagnosed as having invasive ductal carcinoma. The mean tumor size was 2.9 cm (range, 0.9–8 cm), with axillary metastasis in two women (7.1%). Most tumors were triple-negative (78.6%), while six showed low estrogen-receptor positivity (<10%). Ki-67 was <20% in 64.3%, with no high values (≥75%). Three patients received neoadjuvant chemotherapy, with two non-responders. No recurrences occurred during a median follow-up of 51 months. Imaging revealed masses on mammography (85.2%), US (92.9%), and MRI (92.3%), with calcifications in two cases. Most lesions were highly suspicious (BI-RADS 4C or 5) and showed increased vascularity in 92.3% on Doppler US. **Conclusions**: Breast ACC typically presents as a hypervascular, highly suspicious mass. Despite frequent triple-negative profiles, it shows low proliferation, poor response to chemotherapy, and favorable prognosis.

## 1. Introduction

Adenoid cystic carcinoma (ACC) is a variant of adenocarcinoma that mostly occurs in the salivary glands. ACC has been reported to occur in other sites, including the breast, lung, tracheobronchial tree, uterine cervix, larynx, and Bartholin glands [1]. ACC of the breast is rare among breast carcinomas, accounting for less than 0.1% of malignant breast tumors [2]. ACC of the breast is characterized by the presence of both epithelial and myoepithelial cells, which are essential for its pathological diagnosis [1]. The characteristic imaging findings of this neoplasm have not yet been well established. ACC of the breast is classified as a triple-negative breast cancer (TNBC) because most tumors lack expression of estrogen receptor (ER), progesterone receptor (PR), and human epidermal growth factor receptor 2 (HER2) [3]. Compared to other triple-negative breast cancers, ACC of the breast has a favorable prognosis, with low morbidity and mortality rates.

Only a few single-institution studies with follow-up data have investigated ACC of the breast [3,4,5]. To our knowledge, this study represents the largest imaging series of breast ACC reported to date. By comprehensively analyzing clinical and radiological findings, including mammography, breast US, and MRI, we sought to improve understanding of the imaging characteristics of this rare tumor and facilitate its diagnosis and management. Furthermore, analysis of follow-up outcomes may provide additional insight into its clinical behavior and prognosis.

This study aimed to evaluate the clinical features, imaging findings, and follow-up outcomes of patients with ACC of the breast.

## 2. Materials and Methods

### 2.1. Study Population and Clinical Data Acquisition

This retrospective study was approved by the Institutional Review Board of our institution and the requirement for informed consent was waived (IRB No. 2024-01-135-001).

We reviewed the electronic medical records and images of 28 patients who underwent breast cancer surgery for adenoid cystic carcinoma of the breast between February 2010 and December 2023 at the Breast Cancer Center in Samsung Medical Center. Clinical information, including patient characteristics such as age, family history of breast cancer, gene mutations, menopausal status, mode of lesion detection, histological results after biopsy and surgery, adjuvant treatment after surgery, and follow-up outcomes, was reviewed from medical records.

### 2.2. Analysis of Imaging Findings

Preoperative mammography, US, and MRI findings of the tumors were independently reviewed by two breast radiologists (E.Y.K. and E.S.K.) with 21 and 19 years of experience, and discrepancies were resolved by consensus. Imaging findings were categorized based on the Breast Imaging Reporting and Data System (BI-RADS) 5th edition lexicon [6].

All mammography images were full-field digital mammography, all breast US examinations were performed by breast-specialized radiologists, and all breast MRI examinations were performed using a 3.0 T scanner (Achieva scanner, Philips Medical Systems, Best, The Netherlands) with dynamic contrast-enhanced sequences.

### 2.3. Mammographic Features Assessed

Breast composition on mammography was assessed and lesions were divided into mass, mass with calcifications, calcifications only, and focal asymmetry categories when mammographic abnormalities were present.

In mass lesions, the shape (round/oval or irregular), margin (circumscribed or not circumscribed), and density of the lesion (hyperdense or isodense) were analyzed. Presence of architectural distortion was evaluated, and the final BI-RADS assessment category based on the mammographic findings was recorded.

### 2.4. US Features Assessed

On US, background echotexture and the glandular tissue component (GTC) of the breast parenchyma were assessed. For the lesions, we measured the size of the lesion on US, and divided the lesion type into mass and non-mass. In the mass lesions, shape (round/oval or irregular), margin (circumscribed or not circumscribed), orientation (parallel or non-parallel), and echogenicity (hypoechoic or isoechoic) were analyzed. The presence of intra-lesional cysts, calcifications, architectural distortion, and posterior features (absent/enhancement/shadowing) were evaluated. Doppler US was performed for all US-positive lesions. Vascularity was classified as avascular, mildly increased vascularity (one- or two-color Doppler signals), or moderately increased vascularity (three-or-more-color Doppler signals and/or a penetrating feeding vessel). The final BI-RADS assessment category based on the US features was also assessed.

### 2.5. MRI Features Assessed

Background parenchymal enhancement (BPE) on MRI was assessed and classified into minimal, mild, moderate, and marked. All lesions were categorized as either mass or non-mass enhancement. In mass lesions, shape (round/oval or irregular), margin (circumscribed or not circumscribed), internal enhancement pattern (homogeneous or heterogeneous), presence of rim enhancement, and signal intensity on T2-weighted images were analyzed. Lesion size, enhancement kinetics and multiplicity were evaluated in all lesions. Final BI-RADS category was assessed based on the features on breast MRI.

## 3. Results

Clinical and histological findings of 28 patients with ACC are summarized in Table 1.

### 3.1. Clinical Findings

The mean age of the patients was 57 ± 8 years, and all patients were female. Most patients were postmenopausal (82.1%). BRCA mutation results were available in 11 patients, and only one patient tested positive for BRCA 2 mutation. Half of the patients presented with palpable masses, and the other half had screening-detected lesions. Five patients (17.9%) had synchronous ipsilateral breast cancers other than ACC at the time of diagnosis, including two invasive ductal carcinomas (IDCs) and three ductal carcinomas in situ (DCISs). In four patients, the two cancers were located in different quadrants, while in one patient they were located in the same quadrant but were separated by a distance greater than 2 cm. Three patients with ACC alone underwent neoadjuvant chemotherapy (NAC) before surgery. Two were non-responders, and one of them showed disease progression with the development of liver metastasis during the NAC.

The median follow-up period after surgery was 51 months (range, 18–169 months). All patients remained alive without recurrence or distant metastasis except for one patient who died of causes unrelated to breast cancer. Five patients with synchronous ductal carcinoma were all node-negative and also remained alive without recurrence or distant metastasis. The patient who developed liver metastases during NAC remained alive 30 months after diagnosis, with progression of multiple hepatic metastases but no evidence of extrahepatic disease on follow-up CT examinations.

### 3.2. Histological Findings

Preoperative US-guided biopsy was performed in 27 patients, correctly diagnosing ACC in 18 patients (66.7%), whereas 9 patients (33.3%) were misdiagnosed as having IDC. On postoperative pathological examination, the median tumor size was 2.5 cm (range, 0.9–8.0 cm), and the mean tumor size was 2.9 cm. Axillary lymph node metastases were present in two patients (7.1%). Most tumors were triple-negative (22/28, 78.6%). The remaining six tumors were classified as hormone-receptor-positive/HER2-negative, including five tumors with low ER expression (<10%) and one tumor showing heterogeneous ER positivity (4%). Ki-67 was <20% in 18 patients (64.3%), and no tumor demonstrated a high proliferative index (Ki-67 ≥ 75%).

### 3.3. Mammography Findings

Mammography was available for 27 patients, and the findings are summarized in Table 2. Despite the predominance of postmenopausal women (82.1%) and a mean patient age of 57 years, 63.0% of patients had dense breasts on mammography. Mammography demonstrated positive findings in 85.2% of patients (23/27). The mean lesion size on mammography was 2.0 cm (range, 0.8–5.4 cm).

All cases with positive findings showed mass with or without associated suspicious calcifications, and no case manifested as calcifications only. In 23 patients with mammographically positive lesions, 91.3% of patients showed irregular mass with not circumscribed margin. The final mammographic assessment was BI-RADS category 4 or 5 in all cases except one. This lesion appeared as a small, round, circumscribed mass on both mammography and US and was initially classified as BI-RADS category 3 on mammography. However, increased vascularity on Doppler US and rim enhancement on MRI raised suspicion for malignancy, resulting in a BI-RADS category 4A assessment on both US and MRI.

### 3.4. Breast US Findings

**Figure 1:**
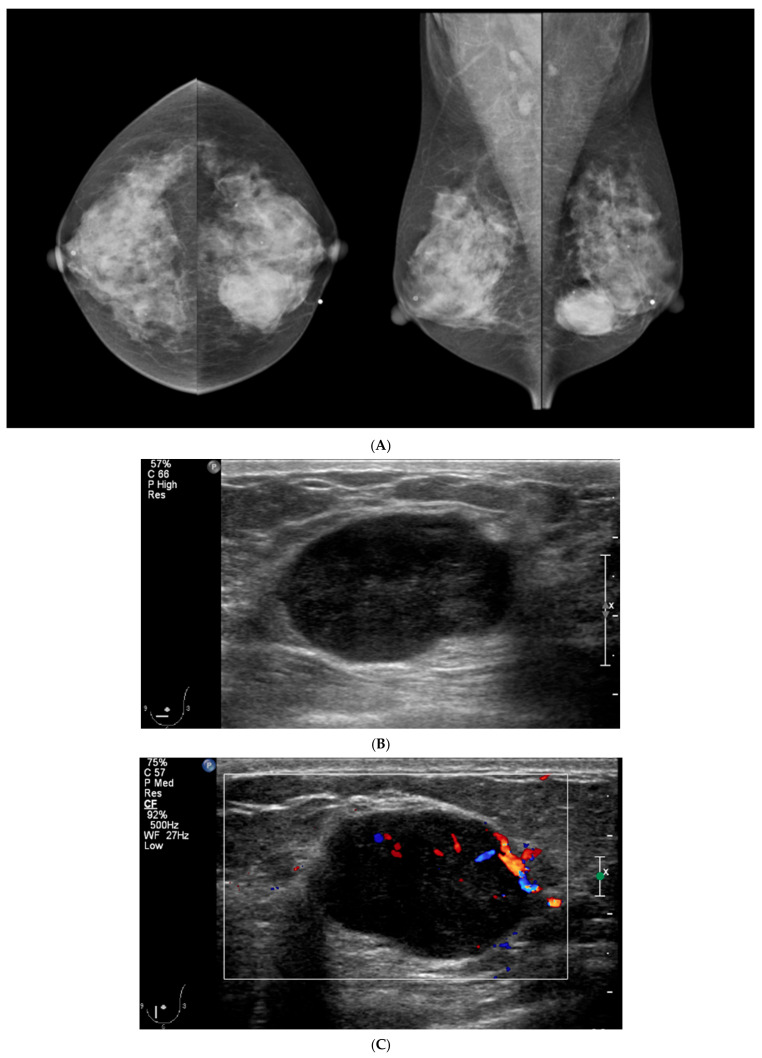

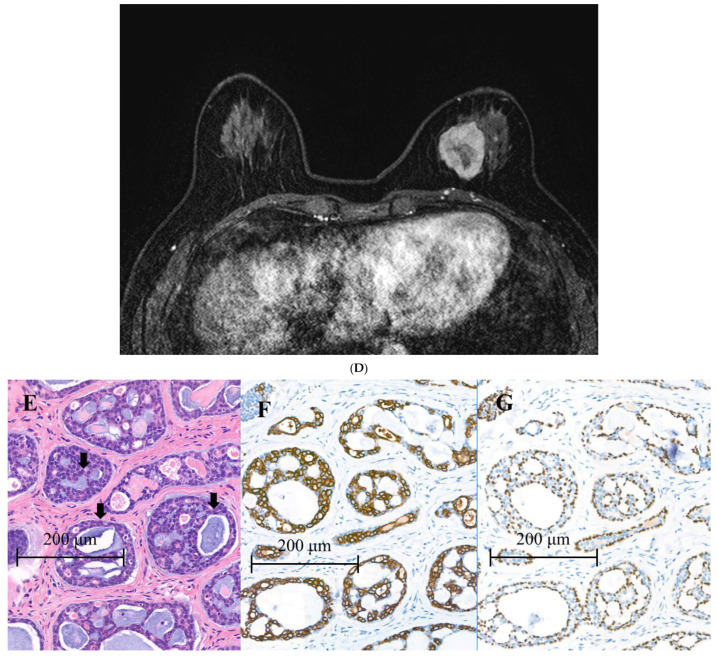
A woman with adenoid cystic carcinoma in the left breast. (**A**) Mammography shows irregular hyperdense mass with a non-circumscribed margin in the lower inner quadrant of the left breast. (**B**) Ultrasound demonstrates an oval-shaped hypoechoic mass with a partially ill-defined margin. (**C**) Doppler ultrasound shows increased intra-tumoral vascularity. (**D**) Breast MR image reveals a 3 × 2.5 cm heterogeneous enhancing mass in the inner breast. (**E**) Histopathology after surgery shows clusters of tumor cells exhibiting a cribriform pattern. Glandular spaces contain mucins (black arrow), suggesting adenoid cystic carcinoma. (**F**,**G**) Immunohistochemistry (IHC) for CK7 (OV-TL 12/30, 1:500, Dako, Glostrup, Denmark) reveals epithelial component of tumor cells, and IHC for p63 (DAK-p63, 1:300, Dako) reveals myoepithelial component of tumor cells.

**Figure 2:**
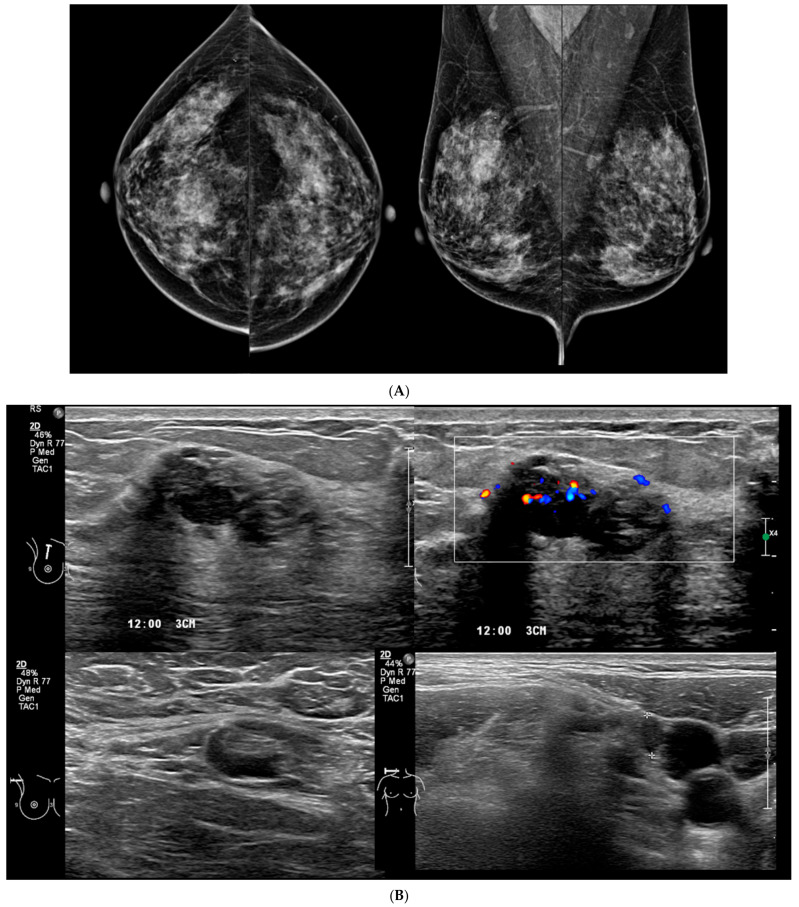

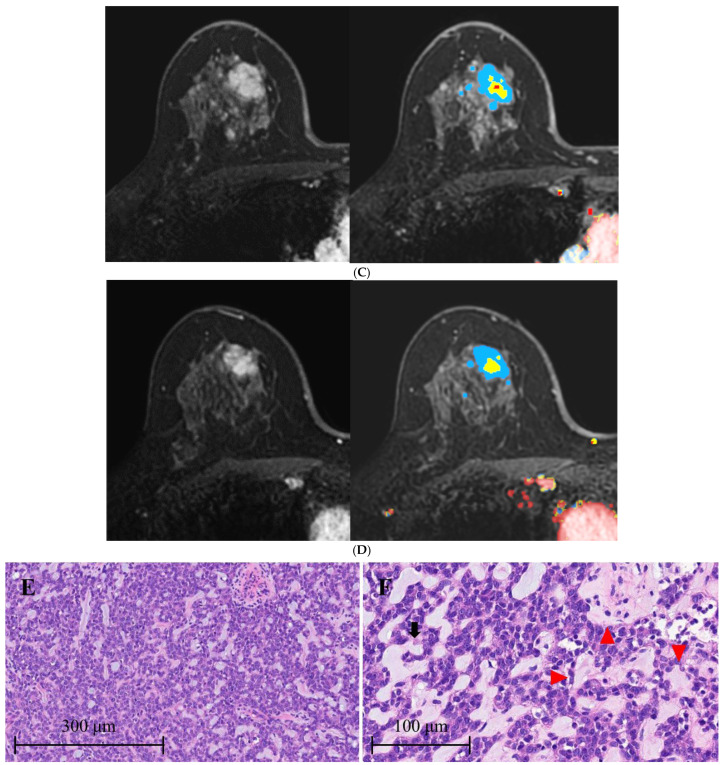
A woman with adenoid cystic carcinoma in the right breast. (**A**) Mammography shows irregular hyperdense mass in the upper center of the right breast. (**B**) Ultrasound shows irregular heterogeneous echoic mass, showing increased vascularity on Doppler ultrasound. Axillary and supraclavicular lymph nodes showed suspicious features, but the results of fine-needle aspiration were negative. (**C**) Breast MR image before neoadjuvant chemotherapy reveals an irregular mass with wash-out kinetics. (**D**) Poor response was found even after neoadjuvant chemotherapy. (**E**) Histopathology after surgery shows clusters of tumor cells arranged in large sheets forming glandular and pseudoglandular structures. (**F**) Glandular structures containing mucin (black arrow) and pseudoglandular structures containing basal membrane and stromal cells (red triangle) suggest the diagnosis of adenoid cystic carcinoma.

The US findings of ACC in the 28 patients are summarized in Table 3. All sonographically detected lesions presented as masses (26/28, 92.9%), most commonly with an irregular shape (65.4%) and non-circumscribed margins (73.1%). Despite their malignant nature, round or oval morphology (34.6%) and circumscribed margins (26.9%) were not uncommon. Among masses with non-circumscribed margins, spiculated or angular margins were uncommon. Instead, many lesions demonstrated partially circumscribed and partially indistinct margins compressing the surrounding structures with expansile growth (57.7%) (Figure 1). Heterogeneous echogenicity, including mixed hypoechoic–hyperechoic or mixed isoechoic–hyperechoic patterns, was observed in eight lesions (30.8%), and six lesions (23.1%) were predominantly isoechoic. More than half of the lesions contained focal isoechoic or hyperechoic components within the mass (Figure 2). No lesion demonstrated an echogenic rind or associated ductal changes.

On Doppler US, increased vascularity was observed in 92.3% (24/26) of lesions. The final BI-RADS assessment was category 4C or 5 in 71.5% of lesions, primarily because of suspicious margins and increased vascularity.

### 3.5. MRI Findings

Preoperative MRI was performed in 26 patients and the findings are summarized in Table 4. All lesions showed positive findings on MRI, and most lesions (24/26, 92.3%) presented as masses. The mean lesion size on MRI was 2.3 cm (range, 0.7–6.0 cm). Mass lesions typically demonstrated highly suspicious morphology, including an irregular shape (87.5%), non-circumscribed margins (87.5%), and heterogeneous internal enhancement (100%). Rim enhancement (16.7%) and high T2 signal (20.8%) were present not uncommonly. Regarding enhancement kinetics, both persistent and wash-out patterns were observed in 12 lesions each (46.2%). The final BI-RADS assessment of MRI was BI-RADS 4C or 5 in 80.8% (21/26).

## 4. Discussion

Adenoid cystic carcinoma (ACC) of the breast is characterized by the presence of a dual cell population of luminal and basaloid cells arranged in specific growth patterns on pathology [7]. ACC features include epithelial cells surrounding hyperchromatic cells with cytoplasm and hyperchromatic nuclei [3]. Histologically, ACC of the breast shows a variety of growth patterns, from small microglandular patterns to solid patterns depending on the area, within the same tumor [8]. The biphasic structure of ACC contributes to the commonly observed cribriform or tubular growth pattern [9]. ACC of the breast presents similar morphological features to the salivary gland tumors, with patterns of tubular–trabecular, cribriform, and solid–basaloid structures. Perineural invasion is a distinctive feature only commonly observed in ACC compared to other breast cancers [10].

In molecular studies, ACC of the breast is often reported as negative for ER, PR, and HER2 expressions, classifying it as triple-negative. However, it demonstrates different characteristics compared to classical basal-cell-like TNBC [11]. In our cohort, patients with ACC were generally older, rarely harbored BRCA mutations, and infrequently exhibited a high proliferative index (>50%) or high histologic grade, features that contrast with those typically associated with basal-like TNBC [12]. This represents the heterogeneity of TNBC, which is not a single specific subtype of breast cancer but rather a group of diverse tumors that only share the common feature of lacking ER, PR and HER2 expression [13,14]. Although ACC frequently demonstrated a triple-negative immunophenotype, it was characterized by low proliferative activity, low histologic grade, poor responsiveness to NAC, and a favorable prognosis. These features may serve as important clues for the preoperative recognition of ACC. Not only in our cohort but also in some previous reports, ACC has been shown to respond poorly to neoadjuvant chemotherapy (NAC) despite its triple-negative phenotype, in contrast to other types of TNBC [15,16]. As seen in our study, ACC is sometimes misdiagnosed as IDC in preoperative percutaneous biopsy, and there is a risk of unnecessary NAC. Therefore, when the percutaneous biopsy results of breast lesions demonstrate triple-negative breast cancer, it is important to differentiate ACC from classical TNBC of the basal-like phenotype based on the clinical and radiological results of our study in order to give patients appropriate personalized treatment. Re-biopsy or large-volume biopsy using a vacuum-assisted device can be considered in these cases.

Despite several differences from classical TNBC, patients with ACC in our cohort presented with relatively large tumors and a high rate of palpable lesions at diagnosis. Compared with the reported symptomatic presentation rate of approximately 34% among breast cancers overall [17], ACC was less frequently detected through screening resembling classical TNBC in terms of the mode of detection [18]. Collectively, these findings suggest that ACC shares certain clinical characteristics with classical TNBC while maintaining distinct biological and clinical features.

Unfortunately, the radiological characteristics are not well known for this rare form of breast cancer. The reported imaging features of adenoid cystic carcinoma are nonspecific and differ widely. In mammography, tumors appear as irregular masses or asymmetry, or benign-looking circumscribed masses [2,19]. US examination presents a hypoechoic or heterogeneous mass with circumscribed or ill-defined margins. Mild peripheral blood flow is presented [19]. Breast MRI reveals an irregular mass with irregular or spiculated margins and heterogeneous enhancement [2,20]. The MRI findings of ACC differ from those of basal-like TNBC that show relatively circumscribed mass with central necrosis, rim enhancement, and wash-out kinetics suggesting the aggressive biology of the tumor [21]. The presence of both luminal and basaloid cells in ACC leads to heterogeneous enhancement on MRI. High signal intensity on T2-weighted images, a feature more commonly associated with benign lesions and mucinous carcinoma, has also been observed in some cases of ACC [2], and was not uncommon in our cases. However, most publications on the radiological findings of breast ACC are case reports of one or two cases [19,20,22].

In our study of 28 cases, most ACCs demonstrated highly suspicious imaging features on both mammography and MRI. In particular, ACC typically presented as an irregular mass with heterogeneous enhancement on MRI, suggestive of malignancy. However, high signal intensity on T2-weighted images was observed in 20.8% of cases, a finding that is uncommon in invasive ductal carcinoma. Compared with IDC, ACC frequently lacked the classic infiltrative appearance of breast malignancy on US. ACC more frequently appeared as a round or oval mass with a parallel orientation, relatively circumscribed but partially indistinct margins, and heterogeneous or isoechoic internal echogenicity. Spiculation, architectural distortion, and calcifications were uncommon, whereas increased vascularity on Doppler US was frequently observed. Unlike classical basal-like TNBC, which often demonstrates cyst-like hypoechogenicity and posterior acoustic enhancement on US [12,21,23], ACC in our study more commonly showed heterogeneous echogenicity (30.8%) and no posterior acoustic features (77.8%). Furthermore, whereas TNBC typically exhibits wash-out kinetics on MRI, reflecting its rapid and aggressive growth pattern [2,5,11,20], 46.2% of ACC cases in our cohort demonstrated persistent enhancement kinetics. Despite the rarity of breast ACC, this study represents the largest single series reported to date, including the largest number of cases with long-term follow-up. The comprehensive radiologic evaluation presented herein, encompassing mammography, breast US, and breast MRI findings, may contribute to a better understanding of the imaging characteristics of this rare tumor and help improve its recognition in clinical practice.

There are a few limitations in our study. First, this is a result from a single institution and the rarity of the disease led to an inevitably small sample size. Our study does not include a statistical analysis comparing ACC with other types of TNBC. Future multi-center studies are warranted to validate our findings and address unsolved questions. Second, we started the patient selection from the list of patients who underwent breast cancer surgery. Percutaneous biopsy-proven ACC without subsequent surgery because of the advanced stage of disease could have therefore been excluded from the study. However, such cases are believed to be exceedingly rare. Third, the radiologists who analyzed the imaging features of ACC were aware that the lesions had been diagnosed as malignant at the time of image review. Therefore, there may have been a tendency to assign higher BI-RADS final assessment categories than would have been assigned in a true diagnostic setting. Finally, there were five patients with synchronous ductal carcinoma at the time of diagnosis in our study population, but the clinical outcomes of those patients were not different from other patients with ACC.

In conclusion, breast ACC typically presents as a hypervascular mass with less infiltrative features on US, despite appearing highly suspicious on mammography and MRI. Although many tumors exhibit a triple-negative immunophenotype, they are characterized by low proliferative activity, limited responsiveness to neoadjuvant chemotherapy, and a favorable prognosis. Recognition of these distinctive clinical, radiologic, and pathologic features may facilitate the accurate diagnosis and appropriate management of this rare breast malignancy.

## Figures and Tables

**Table 1:** Clinical and histological findings of the patients with adenoid cystic carcinoma.

		N = 28 (%)
Family history	present	2 (7.1)
BRCA mutation (n = 11)	positive	1 (9.1)
Menopausal status	premenopausal	5 (17.9)
	postmenopausal	23 (82.1)
Neoadjuvant chemotherapy	underwent	3 (10.7)
Biopsy method	core needle biopsy	25 (89.3)
	vacuum-assisted biopsy	2 (7.1)
	excisional biopsy	1 (3.6)
Biopsy result	adenoid cystic carcinoma	18 (66.7)
	others	9 (33.3)
Operation method	breast conserving surgery	25 (89.3)
	total mastectomy	3 (10.7)
Mean tumor size (range), cm		2.9 (0.9–8)
Axillary node metastasis	present	2 (7.1)
Distant metastasis	positive	1 (3.6)
Tumor grade	low	3 (10.7)
	intermediate	19 (67.9)
	high	3 (10.7)
	unknown	3 (10.7)
Hormone receptor (+)		6 (21.4)
HER2 (+)		0 (0)
Ki67	1	18 (64.3)
	2	8 (28.6)
	3	2 (7.1)
	4	0
Adjuvant chemotherapy	yes	17 (60.7)
Adjuvant radiotherapy	yes	26 (92.9)
Adjuvant hormonal therapy	yes	20 (71.4)
Mode of detection	screening	14 (50)

**Table 2:** Mammographic findings of adenoid cystic carcinoma.

		N = 27 (%)
Density	entirely fatty	1 (3.7)
	scattered fibroglandular	9 (33.3)
	heterogeneously dense	15 (55.6)
	extremely dense	2 (7.4)
Mean lesion size (range), cm		2.1 (0.5–5.4)
Lesion type	negative	4 (14.8)
	mass	21 (77.8)
	mass + calcifications	2 (7.4)
	calcifications only	0
	focal asymmetry	0
Mass (n = 23)		
Shape	oval/round	2 (8.7)
	irregular	21 (91.3)
Margin	circumscribed	2 (8.7)
	not circumscribed	21 (91.3)
Density	hyperdense	14 (60.9)
	isodense	9 (39.1)
AD (n = 23)		2 (8.7)
BI-RADS (n = 23)	3	1 (4.3)
	4A	3 (13.0)
	4B	4 (17.4)
	4C	13 (56.5)
	5	2 (8.7)

AD = architectural distortion.

**Table 3:** Ultrasonographic findings of adenoid cystic carcinoma.

		N = 28 (%)
Background echotexture	homogeneous—fat	5 (17.9)
	homogeneous—fibroglandular	18 (64.3)
	heterogeneous	5 (17.9)
GTC	minimal	13 (46.4)
	mild	13 (46.4)
	moderate	2 (7.1)
	marked	0 (0)
Mean lesion size (range), cm		2.1 (0.7–9)
Lesion type	negative	2 (7.1)
	mass	26 (92.9)
	non-mass	0
Mass (n = 26)		
Shape	oval/round	9 (34.6)
	irregular	17 (65.4)
Orientation	parallel	21 (80.8)
	non-parallel	5 (19.2)
Margin	circumscribed	7 (26.9)
	not circumscribed	19 (73.1)
	spiculated/angular	4 (15.4)
	ill-defined	15 (57.7)
Echo pattern	hypoechoic	11 (42.3)
	isoechoic	6 (23.1%)
	heterogeneous	8 (30.8%)
	complex cystic and solid	1 (3.8)
Calcifications on US	present	1 (3.7)
Architectural distortion	present	1 (3.7)
Posterior feature	no features	21 (77.8)
	enhancement	6 (22.2)
	shadowing	0 (0)
Doppler study	avascular	2 (7.7)
	mildly increased vascularity	7 (26.9)
	moderately increased vascularity	17 (65.4)
BI-RADS	4A	2 (7.1)
	4B	6 (21.4)
	4C	12 (42.9)
	5	8 (28.6)

GTC = glandular tissue component; US = ultrasound.

**Table 4:** MRI findings of adenoid cystic carcinoma.

		N = 26 (%)
BPE	minimal	19 (73.1)
	mild	2 (7.7)
	moderate	4 (15.4)
	marked	1 (3.8)
Mean lesion size (range)		2.3 (0.7–6)
Lesion type	mass	24 (92.3)
	non-mass	2 (7.7)
Mass (n = 24)		
Shape	oval/round	3 (12.5)
	irregular	21 (87.5)
Margin	circumscribed	3 (12.5)
	not circumscribed	21 (87.5)
Enhancement pattern	homogeneous	0 (0)
	heterogeneous	24 (100)
Rim enhancement	present	4 (16.7)
	absent	20 (83.3)
T2	high	5 (20.8)
	not high	19 (79.2)
Enhancement kinetics	persistent	12 (46.2)
	plateau	2 (7.7)
	wash-out	12 (46.2)
Multiplicity	present	4 (15.4)
BI-RADS	3	1 (3.8)
	4A	2 (7.7)
	4B	2 (7.7)
	4C	12 (46.2)
	5	9 (34.6)

BPE = background parenchymal enhancement.

## Data Availability

The datasets generated during and/or analyzed during the current study are not publicly available, but are available from the corresponding author on reasonable request.
